# Copper Modulation as a Therapy for Alzheimer's Disease?

**DOI:** 10.4061/2011/370345

**Published:** 2011-08-23

**Authors:** Yasmina Manso, Gemma Comes, Juan Hidalgo, Ashley I. Bush, Paul A. Adlard

**Affiliations:** ^1^Institute of Neurosciences and Department of Cellular Biology, Physiology and Immunology, Faculty of Biosciences, Autonomous University of Barcelona, Bellaterra, 08193 Barcelona, Spain; ^2^Oxidation Biology Laboratory, The Mental Health Research Institute, Parkville, Vic 3052, Australia; ^3^Synaptic Neurobiology Laboratory, The Mental Health Research Institute, Parkville, Vic 3052, Australia; ^4^Department of Pathology, The University of Melbourne, Parkville, Vic 3010, Australia

## Abstract

The role of metals in the pathophysiology of Alzheimer's disease (AD) has gained considerable support in recent years, with both in vitro and in vivo data demonstrating that a mis-metabolism of metal ions, such as copper and zinc, may affect various cellular cascades that ultimately leads to the development and/or potentiation of AD. In this paper, we will provide an overview of the preclinical and clinical literature that specifically relates to attempts to affect the AD cascade by the modulation of brain copper levels. We will also detail our own novel animal data, where we treated APP/PS1 (7-8 months old) mice with either high copper (20 ppm in the drinking water), high cholesterol (2% supplement in the food) or a combination of both and then assessed **β**-amyloid (A**β**) burden (soluble and insoluble A**β**), APP levels and behavioural performance in the Morris water maze. These data support an interaction between copper/cholesterol and both A**β** and APP and further highlight the potential role of metal ion dyshomeostasis in AD.

## 1. Introduction

Alzheimer's disease (AD) is a progressive neurodegenerative disorder that results in the accumulation and aggregation of key proteins within the brain that are believed to drive the symptomatic presentation and pathological progression of the disease. The underlying cause of AD has yet to be elucidated however, there is an increasing burden of proof on the role of metal dysregulation in the pathogenesis of the disease. Other papers in this special edition will detail the proposed role of copper in the generation and aggregation of key AD-related proteins. In this paper, we will review the preclinical and clinical trial data related to attempts to affect the AD cascade by the modulation of brain copper levels. In addition, we will also present our own novel data on long-term copper administration to APP/PS1 transgenic mice (one of the more widely used mouse models of AD).

## 2. Therapeutic Intervention Studies

In this section, we will outline the various animal and human trials that have been conducted that are specifically directed at a modulation of copper. There are a number of additional studies that will not be discussed, as these assess the effect of therapeutics that may alter a diversity of metals such as copper, zinc, and iron. Keys among these are our own studies with the metal ionophore, PBT2, which has shown promising effects in a number of different transgenic mouse models of AD and also in a phase 2a human clinical trial [1–4]. Its mechanism of action is believed to rely on a normalisation of metal ion (primarily copper and zinc) homeostasis within the brain, which subsequently affects a variety of cellular cascades that, among other activities, serve to facilitate the disaggregation and clearance of beta-amyloid (the principle component of the amyloid plaques that characterises the neuropathology of AD) and also maintains synaptic health and cognition. While these studies are of great value and have provided impressive data to support this avenue of investigation into the treatment of AD, their mechanisms of action are complex and may not necessarily result from an effect on copper alone.

## 3. Preclinical Studies

Utilising the APP23 [B6-Tg(Thy1APP)23SdZ] mouse line, Bayer and colleagues [5] treated aged animals (12 months old) with deionized water containing either sucrose or a combination of sucrose and copper sulfate (250 ppm). This copper sulfate treatment, which lasted for three months, was sufficient to elevate copper levels in the APP23 mice (~25% increase), thereby remedying the homeostatic copper deficit present in this transgenic mouse line. The net effect on amyloid burden was a shift to decreased levels of both PBS-soluble A*β*1-40 and 1-42 in male transgenic mice only. Likewise, formic-acid-soluble amyloid burden was only lower in the male animals treated with copper sulfate, while histological amyloid load was decreased in both the male and female transgenic mice treated with copper sulfate. These data are consistent with our own findings, as reported here. 

**Figure 1:**
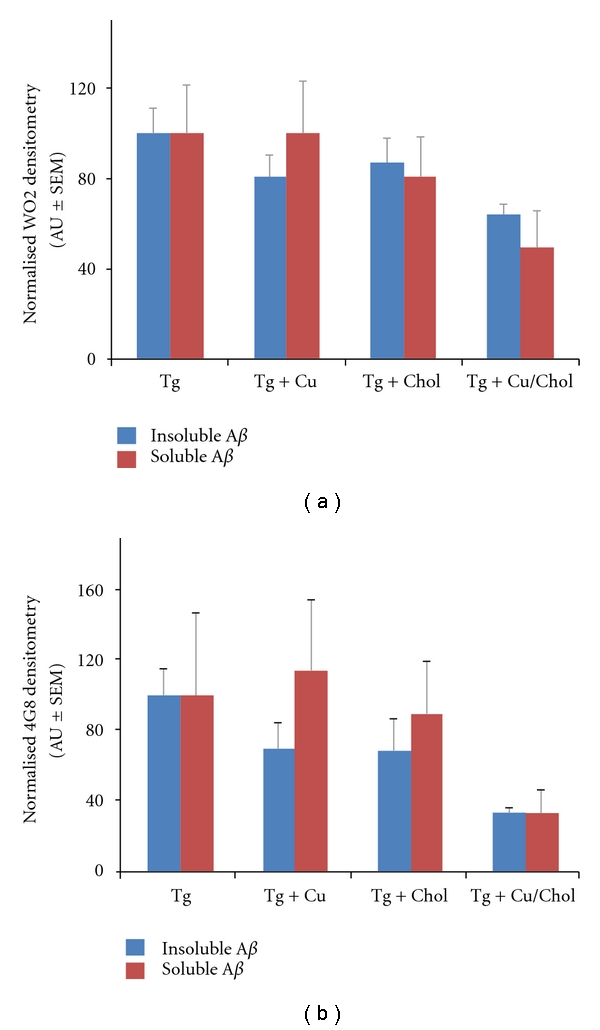
Assessment of A*β* burden in APP/PS1 mice by Western blot. Total hemisphere homogenates (sonicated in PBS followed by centrifugation at 100,000×g for 30 minutes at 4°C and subsequent isolation of the soluble and insoluble fractions. The insoluble fraction was resuspended in PBS prior to a BCA assay and subsequent Western blot) were assessed for A*β* content using both an in-house antibody (WO2) (a) and a commercial (4G8) (b) antibody. A significant reduction in insoluble A*β* was seen with both antibodies for the combined high copper and cholesterol treatment group only, as compared to the Tg control group (Tg).

Utilising APP/PS1 mice (B6C3-Tg(APP_swe_, PSEN1dE9)85Dbo/J on a B6C3 background (stock#004462, The Jackson Laboratory); 7-8 months of age at the start of the trial; animals develop plaques around 4 months of age), we assessed the effect of either copper supplementation in the drinking water (20 ppm in de-ionised water; *n* = 7), cholesterol supplementation in the food (2% cholesterol in regular rodent chow, provided by Specialty Feeds, Western Australia; *n* = 6) or a combination of both (*n* = 4) on the levels of brain amyloid (control animals received regular rodent chow and regular deionised water; *n* = 5). Treatment was for a period of 16 weeks and during the week prior to culling the animals were assessed for spatial learning and memory in the Morris water maze. In this task, animals were subject to six consecutive days of learning trials (4 trials/day/mouse, 90 seconds/trial with a random quadrant entry and a 15 minute inter-trial interval) and one recall task on the seventh day (submerged platform removed from the pool, one 90 second swimming trial/mouse). We have previously published these techniques [3, 6, 7]. The animals were culled the day after the recall task and the tissues collected (trans-cardial PBS-perfusion followed by removal of the brain, which was immediately dissected into two hemispheres, frozen on dry ice and stored at −80°C prior to analysis). Utilising an in-house antibody (WO2), targeted against residues 5–8 of the human A*β* sequence that detects both full-length monomeric A*β* as well as APP species, as well as a commercial A*β* antibody (4G8, residues 17–21) that detects full-length human A*β*, we assessed A*β* burden by western blot. We have previously published these analytical methods [3, 4, 7] and western blot assessments of A*β* have previously been shown to detect the largest “pool” of this peptide in human samples [8]. Our data (Figure 1) demonstrate similar trends for the effect of the individual treatments, with both the elevated copper and the elevated cholesterol diets causing a trend to a decrease in A*β* load. In contrast, a study [9] in a different APP/PS1 model (PS1M146L mice crossed with APPK670N, M671L mice; mice develop plaques at 2-3 months of age) found a trend to an increase in plaque burden in copper-supplemented animals (plaque volume (mm^2^) on distilled water alone: 1.63 ± 0.05; on copper-treated distilled water: 1.84 ± 0.05; *P* = 0.06). This study utilised a different start date for treatment (11 weeks of age, corresponding to the start of plaque formation), period of treatment (6 weeks), dose of copper (0.12 ppm copper sulfate) and method of quantitation (histological assessment of A*β* plaque volume using the antibody, 10D5) than our own work reported here. These variable experimental parameters are likely to account for the differences in the outcomes observed in both studies.

**Figure 2:**
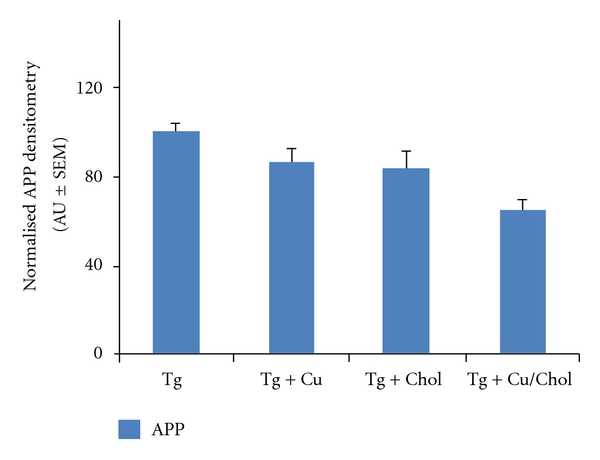
Assessment of APP levels in APP/PS1 mice by Western blot. There is a significant reduction in APP levels in the transgenic mice that received both a high-copper and a high-cholesterol diet, as compared to the Tg control group (Tg).

**Figure 3:**
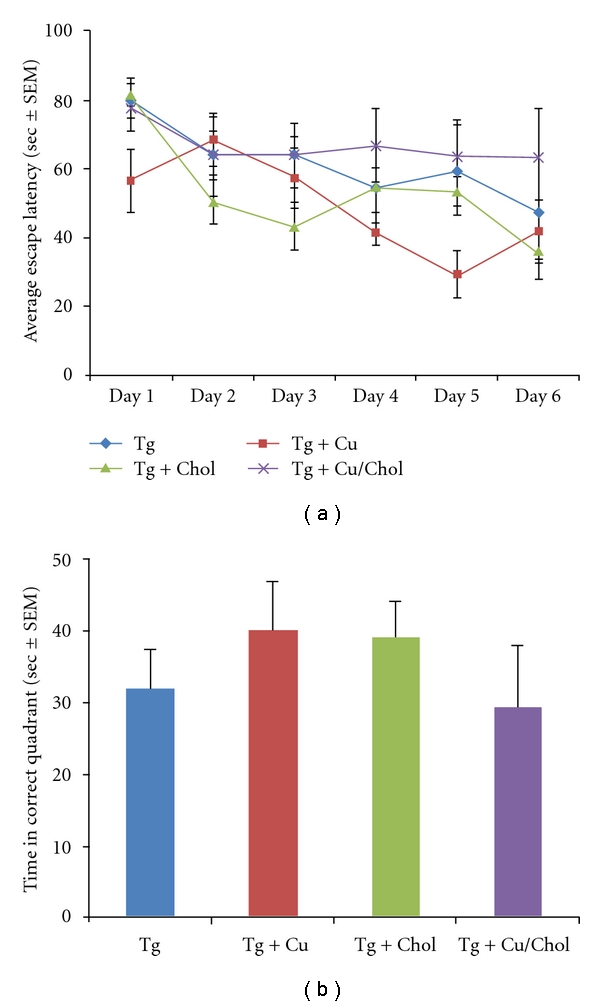
Assessment of spatial learning and memory in APP/PS1 mice in the Morris water maze. There is an overall trend for a modulation of learning performance (a) on this task across the various treatment groups. There was no significant effect on recall in the probe trial across the different treatment groups (b).

The copper and cholesterol treatments utilised in our paradigm also appear to be additive, in that there was a significant and exaggerated decrease in A*β* in the Tg animals that received both elevated copper and cholesterol in their diet, as compared to the transgenic animals receiving the control diet (ANOVA: *P* = 0.02 for WO2 pellet/insoluble data, *P* = 0.007 for 4G8 pellet/insoluble data). This effect was consistent across assays with both the in-house (Figure 1(a)) and the commercial (Figure 1(b)) A*β* antibody. These data suggest an interaction of both copper and cholesterol on A*β* metabolism in this transgenic mouse model of AD. Analysis of APP levels (Figure 2) demonstrated that this reduction in A*β* is likely a function of a direct effect on APP, whose protein expression profile closely paralleled that found for A*β*, with a significant reduction in APP in the animals that received the combined high cholesterol and high copper diet (ANOVA: *P* = 0.0003). There was no significant effect of either the copper treatment (ANOVA: *P* = 0.09) or the cholesterol treatment (ANOVA: *P* = 0.08) on APP levels, although a trend to decrease was observed with both. Despite these changes in A*β* levels, there were no significant effects observed in the performance of animals in the Morris water maze task (Figure 3). Repeated measures ANOVA across the six days of learning trials demonstrated a trend to an overall difference between all treatment groups (*P* = 0.07), with a significant treatment x day interaction (*P* < 0.05). Post hoc analysis revealed a significant difference across the trial when comparing the high-copper treatment and the high-copper/cholesterol treatment groups (repeated measures ANOVA: *P* = 0.02) and a trend to a difference between the high-copper and the control transgenic groups (repeated measures ANOVA: *P* = 0.07). Taken together, these data suggest that the decrease in A*β* resulting from a high-copper diet may improve cognitive function, whereas the combination of both a high-copper and a high-cholesterol diet may negatively impact learning and memory, despite a significant decrease in A*β* burden in those animals. This latter observation, while apparently paradoxical, is consistent with a community-based prospective study that reported that a high-fat diet in conjunction with high-copper intake was associated with a faster rate of cognitive decline in individuals aged 65 years and older [10]. While the mechanisms underlying this have not been pursued, it is likely that an interaction between copper and cholesterol, particularly in the aged brain, may be sufficient to generate unbuffered reactive oxygen species which can contribute to neuronal toxicity and ultimately to cognitive decline [11].

It is also important to note that the effect of elevated copper and/or cholesterol on a “normal” brain may differ markedly to that observed in a “diseased” brain (in which, as is the case in the studies mentioned above, there is an existing homeostatic deficit in copper levels in the brain). This is apparent from a number of studies where the administration of copper and cholesterol to both rabbits [12] and outbred mice [13] resulted in the accumulation of A*β* and an impairment in various learning/memory tasks. 

Thus, more detailed mechanistic investigations into the effect of copper and cholesterol on pathways related both to A*β* metabolism and to cognitive function in both the wildtype and the APP transgenic mouse are required in order to reconcile the differences in these studies.

Following this same principle of elevating copper levels to try and modulate the AD cascade, Phinney and colleagues utilised a genetic approach to achieve this goal [14]. They crossed TgCRND8 mice (a common AD transgenic mouse line that shows a very rapid accumulation of A*β* plaques) with *tx* ^*J*^ mice that harbour an autosomal recessive mutation in the gene encoding the copper transport protein, CuATPase7b. The resulting animals (harbouring both mutant human APP and also homozygous for the ATPase7b mutation) had elevated brain copper levels, and the net effect on amyloid burden was similar to that shown in the two studies above, with a decrease in the total brain levels of both soluble- and formic-acid-extractable A*β* (assessed by ELISA) and a significant reduction in the number of histologically identified dense-cored plaques.

The modulation of brain copper levels, therefore, is sufficient to alter the normal generation and metabolism of A*β* in transgenic mouse models of AD. This hypothesis has been further tested in a number of studies that have pharmacologically manipulated copper levels in the brains of APP transgenic mice.

The copper chelator pyrrolidine dithiocarbamate, for example, was given chronically in the drinking water (20 mg/kg, 7 months) to APP/PS1 mice [15] and resulted in a significant increase in brain copper levels, no significant change to amyloid burden, but did improve spatial memory. In contrast, Crouch and colleagues [16] utilised the copper bis(thiosemicarbazone) complex, CuII(gtsm), which elevates cellular copper concentrations and activates various signalling pathways relevant to amyloid metabolism. When administered to APP/PS1 animals for 15 weeks (10 mg/kg/day), there was a significant decrease in PBS-insoluble amyloid, which appeared to be largely driven by a decrease in A*β* trimer species, and a parallel improvement in performance in the Y-maze memory task.

Thus, these preclinical studies suggest that the modulation of brain copper levels may be sufficient to impact the normal pathogenesis of AD. The translation of this work to human studies, however, has not shown the same potential efficacy of this therapeutic approach.

## 4. Clinical Studies

In the first study of its kind, Squitti and colleagues [17] assessed the effect of the copper-chelating agent, D-penicillamine, (*n* = 17, 600 mg per day for 6 months; *n* = 17 placebo) in a small pilot study in AD patients. A number of cognitive tests were used to assess the effect of the compound, including the Mental Deterioration Battery, MMSE, NeuroPsychiatric Inventory, Geriatric Depression Scale, and the Gottfries Brane Steen scale. While no significant cognitive effects were observed, the placebo-treatment group did not decline as expected, and this precluded any conclusions being drawn on the efficacy of D-penicillamine on cognition (this lack of expected decline in placebo groups has become a recurrent problem in AD trials). Despite this, the data did suggest that there may have been an effect on a number of the biochemical parameters examined. The authors concluded that larger patient numbers were required to further elucidate the potential efficacy of this approach for the treatment of AD.

A more recent clinical trial involved oral copper supplementation in a small cohort of AD patients [18, 19]. Individuals received either placebo (*n* = 33) or oral copper orotate (*n* = 35; equivalent to 8 mg copper per day) for twelve months. The endpoints that were examined included various CSF A*β* species (1-37, 1-38, 1-39, 1-40, and 1-42 species), tau and phospho-tau (thr181) levels and cognitive function, assessed using the Alzheimer's Disease Assessment Scale (ADAS-cog) and the minimental state examination (MMSE). The only biomarker that changed was A*β*1-42 levels, which decreased less (10% drop) in the copper-supplementation group over the course of the study, as compared to the placebo group (30% drop). All other biomarkers and the cognitive scores were unchanged between groups. The implications of the effect seen on CSF amyloid burden are unclear, and the authors concluded that the copper supplementation had no effect on the progression of the AD phenotype.

## 5. Conclusions

These data highlight the difficulty in translating basic bench science into effective therapeutics. While it is clear that there is a dyshomeostasis in copper levels in the AD brain that may contribute to the pathogenesis of AD (both through a disruption to normal copper-dependent pathways and also via an effect of copper on the aggregation and toxicity of amyloid and formation of plaques) and that in vivo models support the use of compounds that are able to modulate copper levels as being effective at interfering in the normal AD cascades, testing these notions in a human population have thus far been lacking. A definitive understanding of the mechanisms of action and potential interactions of copper in the AD brain are also lacking and are clearly complex. Metals such as copper, for example, are very tightly regulated, and in situations where there is a mismetabolism of copper, a simple dietary supplement is unlikely to change this mismetabolism and may not result in a restoration of cellular copper homeostasis. The “unregulated” delivery of copper to the AD brain may, for example, result in a number of consequences: it may potentiate the aggregation and accumulation of *β*-amyloid deposits (which arguably may represent either a toxic or a protective phenomenon, depending on ones belief about the role of and interaction between soluble oligomers and insoluble A*β* plaques in the pathogenesis of AD); it may aggravate oxidative pathways to generate toxic ROS or also have other negative cellular effects; it may also activate a number of intracellular copper-dependent pathways that mediate an improvement in multiple aspects of the pathophysiology of AD, including facilitating the degradation of A*β* and reducing the abnormal phosphorylation of tau (as has been shown when using a more targeted pharmacological approach for the normalisation of copper homeostasis, such as CuII(gtsm) reviewed in [20]). It is clear, therefore, that attempting to modulate homeostatic systems is fraught with difficulties and will require sophisticated approaches to the targeted restoration of metal levels within the brain to ensure that the required outcomes are achieved in the absence of any toxicity. A greater burden of proof, and the identification of a candidate compound, is required to help move this avenue of research forward into robust human clinical trials.
